# Microarray and ChIP-seq data analysis revealed changes in p53-mediated transcriptional regulation in Nutlin-3-treated U2OS cells

**DOI:** 10.3892/mmr.2015.3933

**Published:** 2015-06-15

**Authors:** SONG ZHAO, FENG NIU, CHANG-YAN XU, LONG YE, GUI-BIN BI, LIN CHEN, PING GONG, GANG TIAN, TIAN-HONG NIE

**Affiliations:** 1 Department of Spinal Surgery, The First Hospital of Jilin University, Changchun, Jilin 130021, P.R. China; 2 Department of Medical Records, The First Hospital of Jilin University, Changchun, Jilin 130021, P.R. China

**Keywords:** p53, osteosarcoma, transcription factor, chromatin immunoprecipitation-sequencing data, differentially expressed genes, functional enrichment analysis

## Abstract

Integrative analysis of chromatin immunoprecipitation-sequencing (ChIP-seq) data and microarray data was performed to illustrate the effect of Nutlin-3 on promoter selectivity and transcriptional regulation by the tumor suppressor p53 in U2OS human osteosarcoma cells. Raw data (accession number, GSE46642) were downloaded from Gene Expression Omnibus. Differential analyses were performed using package limma of *R* software. Gene ontology enrichment and Kyoto Encyclopedia of Genes and Genomes pathway enrichment analyses were performed for the differentially expressed genes (DEGs) using the Database for Annotation, Visualization and Integration Discovery. Integrative analysis of ChIP-seq data and microarray data were confirmed with ChIP-Array. A total of 565 DEGs were identified, including 373 upregulated genes and 192 downregulated genes. Genes involved in the p53 signaling pathway, cell cycle, DNA replication, cytokine-cytokine receptor interaction and melanoma were markedly over-represented in the DEGs. A total of 39 DEGs were directly regulated by p53 and two were the transcription factors (TFs), E2F2 and HOXA1. E2F2 regulated 25 DEGs, while HOXA1 regulated one DEG. The cell cycle, p53 signaling pathway, melanoma and pathways involved in cancer were enriched in the direct and indirect target genes. Changes in the p53-binding pattern induced by Nutlin-3 were described in the present study, which may advance the understanding of the regulatory network of p53 in osteosarcoma and aid in the development of novel therapies.

## Introduction

Gene transcription is regulated by dynamic interactions between *cis*-regulatory elements and regulatory proteins, including transcription factors (TFs). Tumor protein p53 is an important TF involved in various cellular processes, including growth arrest, senescence and apoptosis (1–3). Following cellular stress, stabilized p53 translocates into the nucleus and subsequently binds to the consensus sequence motif to regulate the expression of hundreds of genes.

p53 is critical in tumor suppression and loss of p53 function is required for cancer progression. Mutational inactivation of p53 is detected in >50% of human cancer types (4). A number of downstream proteins of p53 have been identified (5–7). Nevertheless, several of the factors expected to affect p53-induced changes in gene expression are poorly understood, including the impact of different stresses that can induce p53. Genome-wide studies may provide an improved understanding of its transcriptional regulatory functions in certain types of cancer (8–10), including osteosarcoma.

Osteosarcoma is the eighth most common type of childhood cancer and is also the most common histological form of primary bone cancer (11). The mortality rates for osteosarcoma have been declining by ~1.3% annually (12). The overall 5-year survival rate for osteosarcoma is ~68% (12). Future studies are required to fully disclose the molecular mechanisms and advance therapeutic development.

In the present study, human U2OS osteosarcoma cells, expressing wild-type p53, were used to investigate the effect of treatment with Nutlin-3 (a non-genotoxic activator of p53) on p53 binding genes. Different from a previous study by Menendez *et al* (13), a stricter threshold [|log_2_fold change (FC)|>1 and false discovery rate (FDR) <0.05 vs. FC>2 and FDR ≤0.1] was used to select the differentially expressed genes (DEGs) and to construct the regulatory association between p53 and its target genes.

## Materials and methods

### Raw data

The raw data (accession number, GSE46642) were downloaded from Gene Expression Omnibus (http://www.ncbi.nlm.nih.gov/geo/), including chromatin immunoprecipitation-sequencing (ChIP-seq) data (accession number, GSE46641; three Nutlin-3 treated U2OS cell samples) and microarray data (accession number, GSE46493; three Nutlin-3 treated U2OS cell samples and three control samples). Gene expression levels were measured using Affymetrix Human Genome U133 Plus 2.0 Array (Affymetrix Inc., Santa Clara, CA, USA).

### Pre-treatment and differential analysis

The microarray data were read using the package, affy (14), on the software *R* (http://www.r-project.org/). Following background correction and normalization with a Robust Multi-array Analysis (RMA) method in *R* affy, the gene expression levels were determined. Differential analysis was performed using the package, linear models for microarray data (limma) (15), on the software *R*. Multiple-testing correction was performed using the Bayes method (implemented in the 'limma' *R* package). The following threshold was set for the screening of the DEGs: |log_2_ FC|>1 and FDR<0.05.

### Integrative analysis of microarray data and ChIP-seq data

ChIP-Array (http://jjwanglab.org/chip-array) is an online tool developed for integrative analysis of microarray data and ChIP-seq data (16). It identifies the indirect target, Z, by identifying an intermediate transcription factor (TF), Y, which is a putative regulator of Z and a target of X. The putative regulator of Z is identified by scanning all promoters in the genome with position weight matrix (PWMs) of all Ys from three publicly accessible databases [JASPAR (http://jaspar.genereg.net), UniPROBE (http://uniprobe.org) and TRANSFAC (http://www.gene-regulation.com/pub/databases.html) derived transcription factor binding site database from University of California, Santa Cruz genome browser] (16).

In the present study, the parameters were set as follows: Promoter range, −500~+100; TF database, UniPROBE; PWM scan P-value, 10^−5^; and conservation filtering P-value, 0.001. Finally, a gene regulatory network was obtained for p53, including its direct and indirect target genes.

### Functional enrichment analysis

Gene Ontology (GO) and Kyoto Encyclopedia of Genes and Genomes (KEGG) pathway enrichment analyses were performed for the DEGs using the Database for Annotation, Visualization and Integration Discovery (http://david.abcc.ncifcrf.gov/) online tools (17). P<0.05 was considered to indicate a statistically significant difference and was set as the cut-off.

## Results

### Differentially expressed genes

**Figure 1:**
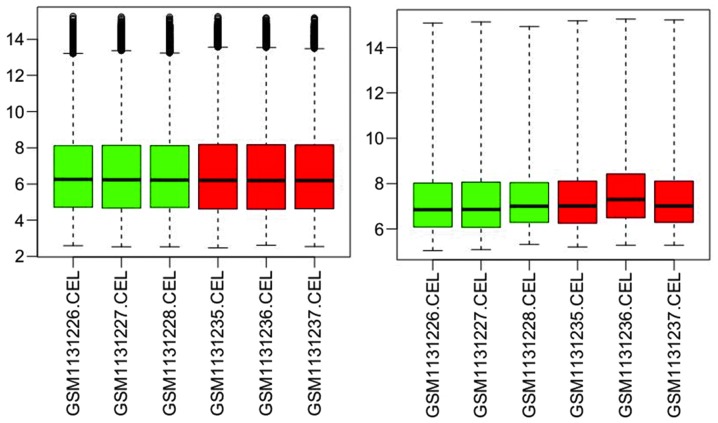
Box plots of gene expression data prior to (left) and following normalization (right). Nutlin-3 treated U2OS samples are shown in red and control samples are in green.

Gene expression data prior to and following normalization with the RMA method are demonstrated in Fig. 1. A good performance of normalization was achieved.

**Figure 2:**
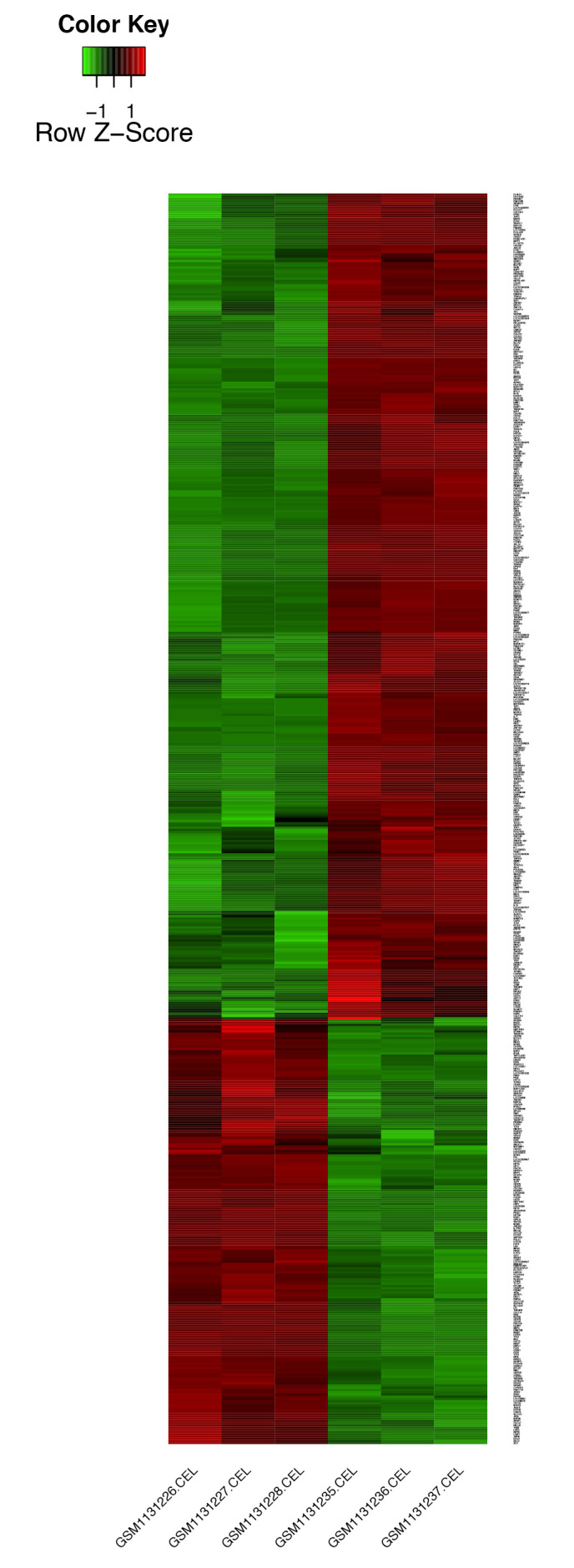
Clustering and heatmap of expression values for differentially expressed genes. Downregulated genes are shown in green and upregulated genes are in red. From left to right, the first three samples are nutlin-3-treated U2OS samples and the latter are three control samples.

A total of 565 DEGs were identified, including 373 upregulated genes and 192 downregulated genes. Clustering and a heat-map of the expression values for DEGs are shown in Fig. 2. The Nutlin-3 treated U2OS samples were well distinguished from the control samples, suggesting the reliability of the DEGs.

### Functional enrichment analysis result

**Figure 3:**
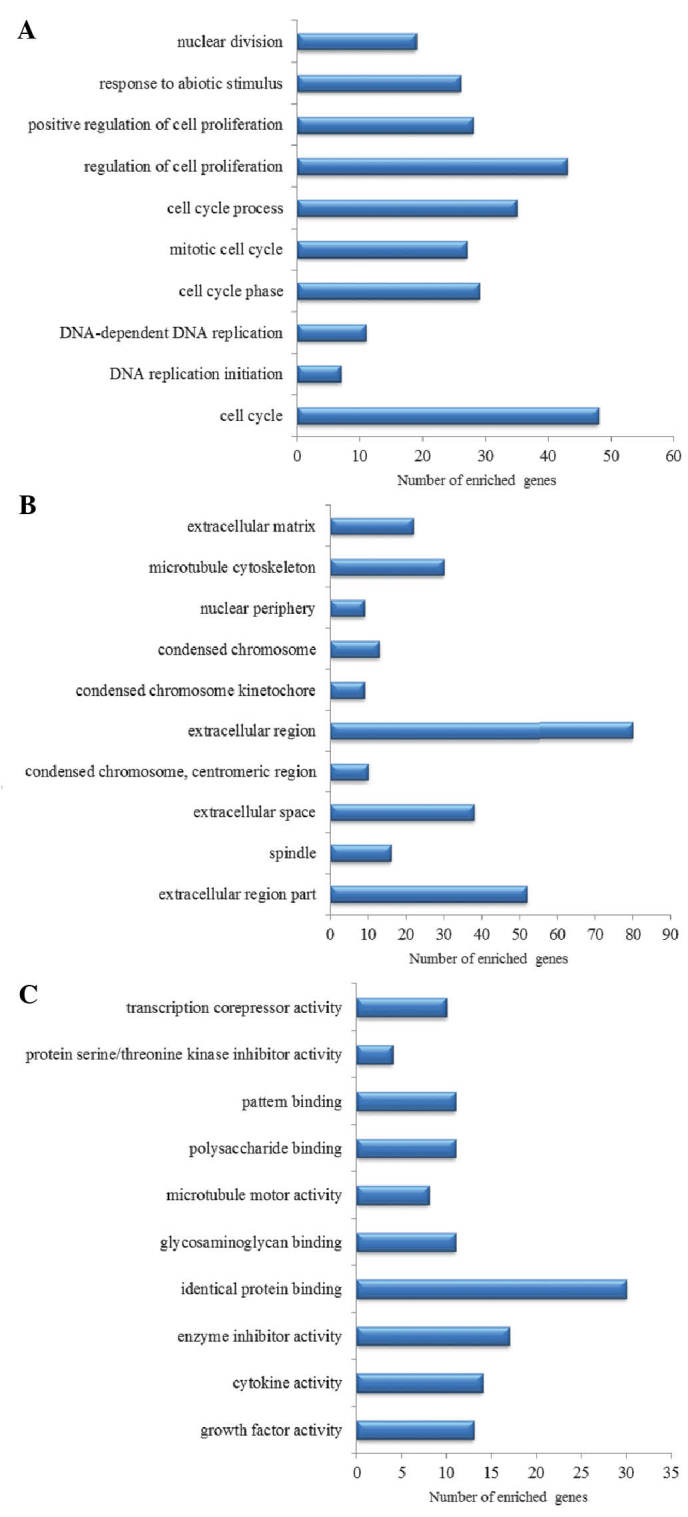
Gene ontology term enriched in the differentially expressed genes. (A) Biological process, (B) cellular component and (C) molecular function.

The top 10 GO terms are listed in Fig. 3. Nuclear division, the response to abiotic stimulus, positive regulation of cell proliferation and cell cycle were significantly enriched in the DEGs.

The KEGG pathways with P<0.05 are listed in Table I. The p53 signaling pathway, cell cycle, DNA replication, cytokine-cytokine receptor interaction and melanoma were significantly over-represented in the DEGs.

### Transcriptional regulatory network of p53

**Figure 4:**
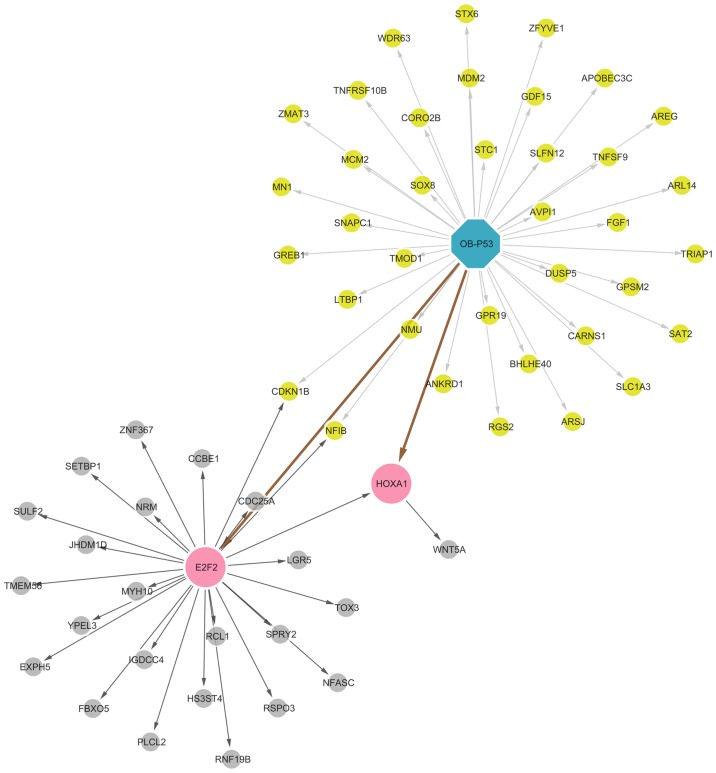
Transcription regulatory network of p53. p53 is shown in blue and its direct target genes are in yellow. Two transcription factors are shown in pink and their target genes are in grey. E2F2, E2F transcription factor 2; HOXA1, homeobox A1.

Integrative analysis of ChIP-seq data and microarray data was performed using the ChIP-Array online tool. A total of 39 DEGs were directly regulated by p53, and two of them were TFs: E2F transcription factor 2 (E2F2) and homeobox A1 (HOXA1). E2F2 regulated 25 DEGs and HOXA1 regulated one DEG (Fig. 4).

### Functional enrichment analysis result of the target genes

**Figure 5:**
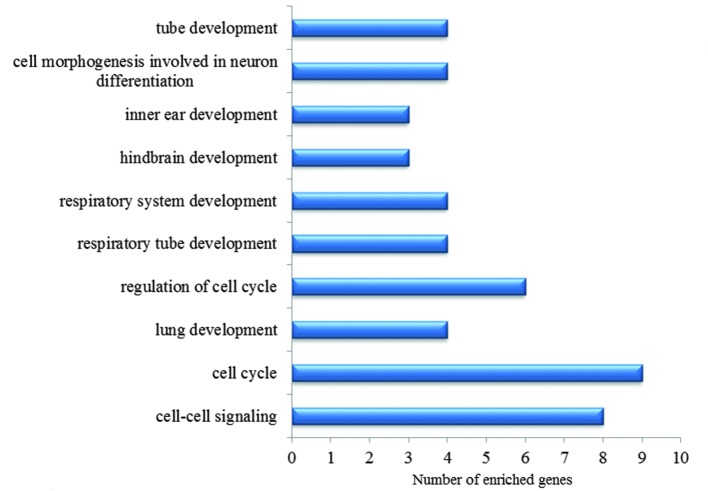
Gene ontology biological process terms enriched in the direct and indirect target genes of p53.

GO enrichment analysis was performed for the direct and indirect target genes of p53 (Fig. 5). Cell cycle and cell-cell signaling were included in the list.

The KEGG pathway enriched in all the target genes of p53 were also disclosed (Table II), including cell cycle, p53 signaling pathway, melanoma and pathways in cancer.

## Discussion

In the present study, a total of 565 DEGs were identified in Nutlin-3-treated U2OS cells compared with the control samples. Of these DEGs, 373 were upregulated genes and 192 were downregulated genes. Functional enrichment analysis revealed that the p53 signaling pathway, cell cycle and DNA replication were significantly over-represented in the DEGs. This result suggested the importance of p53 in osteosarcoma. p53 functions as a cell cycle control protein in osteosarcoma (18) and the presence of p53 mutations in human osteosarcoma is correlated with high levels of genomic instability (19), confirming the critical importance of p53 in response to stresses, including DNA damage. Berman *et al* (20) reported that metastatic osteosarcoma is induced by the inactivation of Rb and p53 (20). The comparative analysis of gene expression profiles between Nutlin-3-treated U2OS cells and controls further described the critical importance of p53 in osteosarcoma. Notably, p53 gene therapy of human osteosarcoma is also suggested and has been previously investigated (21).

To further illustrate the changes in the p53-binding pattern in response to treatment with Nutlin-3, integrative analysis of microarray data and ChIP-seq data was performed and the transcriptional regulatory network of p53 was obtained. A total of 39 DEGs were directly regulated by p53 and two of which were the TFs, E2F2 and HOXA1. E2F2 regulated 25 DEGs and HOXA1 regulated only one DEG. Functional enrichment analysis demonstrated that the cell cycle, p53 signaling pathway, melanoma and pathways in cancer were enriched in the direct and indirect target genes, further confirming the critical importance of p53 in osteosarcoma. It may be beneficial to further investigate these target genes to reveal the complete molecular mechanisms and provide potential therapeutic targets.

Several direct target genes of p53 have been confirmed to be involved in tumorigenesis. The MDM2 proto-oncogene is a nuclear-localized E3 ubiquitin ligase. MDM2 promotes tumor formation by targeting p53 for proteasomal degradation (22). The gene is itself transcriptionally regulated by p53. Therefore, targeting the p53-MDM2 interaction is hypothesized as a cancer therapeutic agent (23,24). Syntaxin 6 (STX6) is a regulator of the protein trafficking machinery. Zhang *et al* (25) indicated that STX6 is an effector and a modulator of the p53 family in the regulation of cell adhesion and survival. Fibroblast growth factor 1 (FGF1) is a member of the FGF family. Bouleau *et al* (26) indicated that FGF1 inhibits p53-dependent apoptosis and cell cycle arrest via an intracrine pathway. Meningioma 1 stimulates vitamin D receptor-mediated transcription and inhibits osteoblast cell proliferation (27). It is required for appropriate osteoblast proliferation, motility, differentiation and function (28). The present study hypothesized that this protein may be a novel target to modulate osteosarcoma cell growth.

E2F2 and HOXA1 are directly targeted by p53 and they are also TFs. It has been confirmed that E2F2 inhibits tumorigenesis (29,30). E2F activity is critical for the control of the G1 to S phase transition. Laresgoiti *et al* (31) demonstrated that E2F2 and CREB cooperatively regulate the transcriptional activity of cell cycle genes. Cyclin-dependent kinase inhibitor 1B (CDKN1B) is one of the effectors of E2F2, which is important in the cellular transition from quiescence to the proliferative state. HOXA1-stimulated oncogenicity is mediated by selective upregulation of components of the p44/42 MAP kinase pathway in human mammary carcinoma cells (32). The expression level of HOXA1 is correlated with poor prognosis of oral squamous cell carcinoma (33). The only TF of HOXA1 is wingless-type MMTV integration site family member 5A (Wnt5a). It is reported that Wnt5a signaling is involved in the regulation of osteosarcoma cell invasiveness (34).

In conclusion, differential expression of several direct and indirect target genes of p53 was observed following treatment with Nutlin-3. These findings not only advanced the understanding regarding the importance of p53 in osteosarcoma, but also provided clues for future development of therapeutic strategies.

## Figures and Tables

**Table I:** Kyoto Encyclopedia of Genes and Genomes pathways enriched in the differentially expressed genes.

Pathway	Count	P-value	Genes
p53 signaling pathway	13	2.49E-07	ZMAT3, RRM2B, SESN1, GTSE1, TP53I3, CDKN1A, TNFRSF10B, BBC3, SERPINB5, SERPINE1, MDM2, FAS, GADD45A
Cell cycle	16	1.39E-06	E2F2, MAD1L1, ESPL1, MCM2, MCM3, MCM4, CDC25A, MCM5, MCM6, CDKN1A, CDC45, CDKN1B, CDKN2C, BUB1B, MDM2, GADD45A
DNA replication	8	4.62E-05	PRIM1, POLD4, POLA1, MCM2, MCM3, MCM4, MCM5, MCM6
Cytokine-cytokine Receptor interaction	17	2.23E-03	TNFSF4, IL7, IL18, KITLG, TNFRSF14, TNFSF9, IL11, IL17RB, CCL26, TSLP, TNFRSF10B, CCL20, CXCL14, INHBE, CCL3L3, PDGFC, FAS
Melanoma	7	1.33E-02	E2F2, FGF5, CDKN1A, FGF9, MDM2, PDGFC, FGF1

**Table II:** Kyoto Encyclopedia of Genes and Genomes pathways enriched in the direct and indirect target genes of p53.

Pathway	Count	P-value	Genes
Cell cycle	5	1.01E-03	E2F2, CDKN1B, MDM2, MCM2, CDC25A
p53 signaling pathway	3	2.60E-02	TNFRSF10B, ZMAT3, MDM2
Melanoma	3	2.82E-02	E2F2, MDM2, FGF1
Pathways in cancer	5	3.04E-02	WNT5A, E2F2, CDKN1B, MDM2, FGF1
Chronic myeloid leukemia	3	3.12E-02	E2F2, CDKN1B, MDM2
